# Advancing surgical VQA with scene graph knowledge

**DOI:** 10.1007/s11548-024-03141-y

**Published:** 2024-05-23

**Authors:** Kun Yuan, Manasi Kattel, Joël L. Lavanchy, Nassir Navab, Vinkle Srivastav, Nicolas Padoy

**Affiliations:** 1 University of Strasbourg, CNRS, INSERM, ICube, UMR7357 Strasbourg, France; 2 IHU, Strasbourg, France; 3 CAMP, Technische Universität München, Munich, Germany

**Keywords:** Visual question answering, Multi-modality learning, Surgical data science

## Abstract

**Purpose:**

The modern operating room is becoming increasingly complex, requiring innovative intra-operative support systems. While the focus of surgical data science has largely been on video analysis, integrating surgical computer vision with natural language capabilities is emerging as a necessity. Our work aims to advance visual question answering (VQA) in the surgical context with scene graph knowledge, addressing two main challenges in the current surgical VQA systems: removing question–condition bias in the surgical VQA dataset and incorporating scene-aware reasoning in the surgical VQA model design.

**Methods:**

First, we propose a surgical scene graph-based dataset, SSG-VQA, generated by employing segmentation and detection models on publicly available datasets. We build surgical scene graphs using spatial and action information of instruments and anatomies. These graphs are fed into a question engine, generating diverse QA pairs. We then propose SSG-VQA-Net, a novel surgical VQA model incorporating a lightweight Scene-embedded Interaction Module, which integrates geometric scene knowledge in the VQA model design by employing cross-attention between the textual and the scene features.

**Results:**

Our comprehensive analysis shows that our SSG-VQA dataset provides a more complex, diverse, geometrically grounded, unbiased and surgical action-oriented dataset compared to existing surgical VQA datasets and SSG-VQA-Net outperforms existing methods across different question types and complexities. We highlight that the primary limitation in the current surgical VQA systems is the lack of scene knowledge to answer complex queries.

**Conclusion:**

We present a novel surgical VQA dataset and model and show that results can be significantly improved by incorporating geometric scene features in the VQA model design. We point out that the bottleneck of the current surgical visual question–answer model lies in learning the encoded representation rather than decoding the sequence. Our SSG-VQA dataset provides a diagnostic benchmark to test the scene understanding and reasoning capabilities of the model. The source code and the dataset will be made publicly available at: https://github.com/CAMMA-public/SSG-VQA.

**Supplementary Information:**

The online version contains supplementary material available at 10.1007/s11548-024-03141-y.

## Introduction

**Fig. 1:**
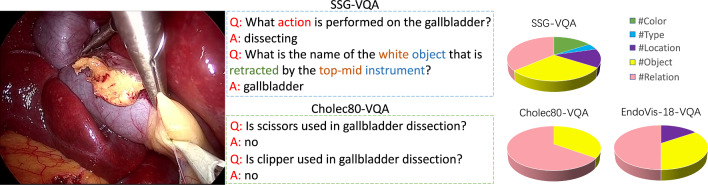
SSG-VQA dataset contains up to 50 complex visual reasoning questions, compared to 2 classification-based questions in the Cholec-VQA [8]

Surgical data science is a rapidly growing field that aims to streamline clinical workflows and enable the development of real-time intra-operative decision-support systems [1, 2]. Recent advancements in surgical video analysis, such as surgical workflow phase recognition, fine-grained surgical action detection and surgical semantic scene segmentation, show evidence of the progress [3–5]. However, the scope of these methods is confined as it mainly focus on visual-only data to perform classification or recognition tasks, thereby offering limited user interaction. The next generation of surgical data science applications also demands approaches operating at the crucial intersection of vision and language to offer intuitive user interaction during intra-operative surgical procedures. Surgical visual question answering (VQA) is emerging as a notable solution in that direction, which aims to provide precise answers to user queries in a natural language by analyzing a given surgical image [6–9].

Developing an effective surgical VQA system is inherently challenging for a typical surgical scene, which contains multiple anatomical structures and instruments connected through diverse spatial and action relationships. While a few works have explored VQA tasks in the surgical context [8, 9], they are typically limited to datasets and models that ignore detailed scene knowledge. From the dataset perspective, one key challenge is the lack of a dataset with potentially a vast set of question–answer pairs covering various aspects of the surgical scene. The current surgical VQA datasets are small and only consider simple scene information, e.g., object/action occurrence, as shown in Fig. 1. Moreover, these datasets contain question–answer pairs with significant question–condition bias, where answers can be derived from just the questions without performing any visual processing. This hinders the utility of these datasets to serve as appropriate surgical VQA benchmarks. From the model perspective, the current surgical VQA architectures operate on the global visual representation of the surgical image, ignoring the detailed understanding of surgical scene knowledge. This can be detrimental, especially when object-level visual reasoning is essential to answer fine-grained questions. Our key contributions are therefore twofold: the introduction of a new surgical scene-aware VQA dataset called SSG-VQA and a novel surgical VQA model called SSG-VQA-Net.

The SSG-VQA dataset uses a semantic scene graph [10] as a suitable representation to generate diverse question–answer pairs. A semantic scene graph representation provides scene knowledge by detecting objects and their attributes and connecting relationships and interactions between objects in the scene. To develop the surgical semantic scene graph, we use publicly available datasets for semantic segmentation and tool detection [11, 12], and apply these models to estimate object spatial relationships. We then estimate the surgical action relationships, i.e.,< *instrument*, *verb*, *target*>, among the objects using the public CholecT45 dataset [3].

**Fig. 2:**
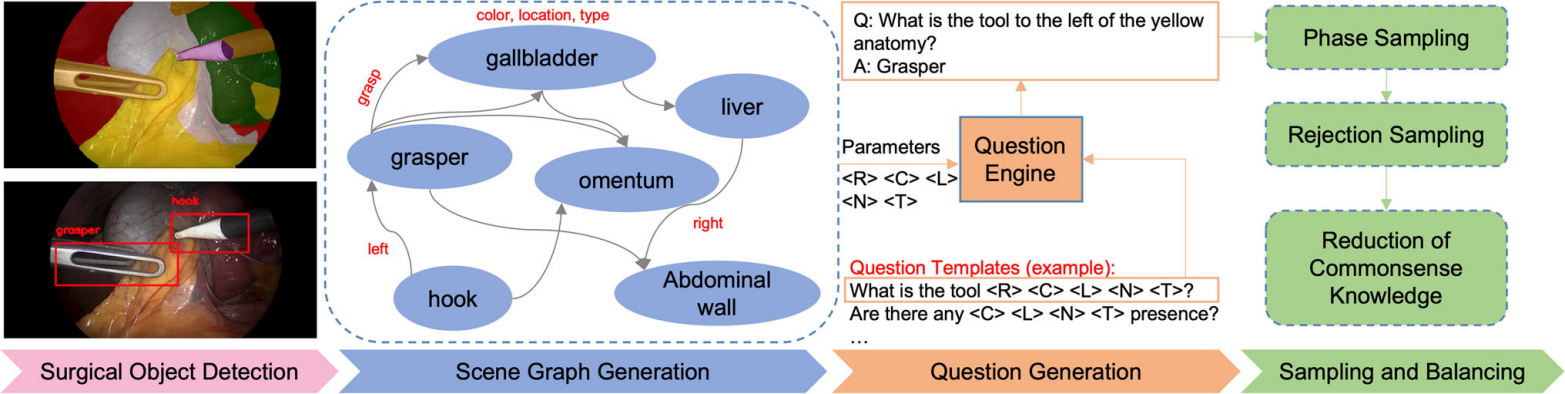
Pipeline of SSG-VQA construction. The dataset is constructed from the well-designed question engine, which takes the scene graph as input and changes the parameters of question templates to generate diverse question–answer pairs

We then develop a surgery-specific question engine that ingests the surgical scene graph and manually designed question templates to produce a variety of question–answer pairs. Including detailed surgical scene understanding along with question templates helps us to generate question–answer pairs covering various aspects of the surgical scene, for example, fine-grained action recognition—“What is the action being performed on peritoneum?”, semantic scene reasoning—“What anatomy is at the top-mid of the frame?”, and surgical object attribute reasoning—“What is the name of the anatomy that is being retracted?”. Furthermore, we perform the balancing and sampling strategies based on the surgery-specific knowledge and class distribution to remove the questions that contain question–condition bias, e.g., “How many livers are in the frame?” which counts the number of certain anatomical structures. The overall pipeline is illustrated in Fig. 2.

Given a large-scale SSG-VQA dataset containing fine-grained surgical question–answer pairs, we propose a multi-modality surgical VQA model called SSG-VQA-Net. Existing surgical VQA models use a highly parameterized multi-modality transformer encoder to fuse the textual embeddings from a question and the patches from a global visual representation of a surgical image [8, 9]. However, these patches do not contain object-wise information about the surgical scene, hence missing the geometric scene understanding. Our key idea is to exploit object-wise local features and fuse geometric scene information in the VQA model design. To enable this, we train a fast and lightweight object detector, YOLOv7 [13], on the bounding box labels of the SSG-VQA dataset. The trained object detector allows us to extract object-wise local representations of the surgical scene objects using RoIAlign pooling [14]. Furthermore, we integrate the geometric spatial coordinates and class labels of detected bounding boxes into the VQA model by introducing a lightweight multimodal transformer encoder named the Scene-embedded Interaction Module (SIM). The SIM uses a scene graph of detected bounding boxes where each node contains the class label and bounding box coordinate information. The scene graph is refined by cross-attention between the scene graph and the textual inputs, highlighting specific graph nodes correlated with the complex question query. These refined text-aware scene embeddings are then combined with the object-wise local representations of the surgical scene and the textual embeddings through a transformer encoder layer to generate an accurate response. Experimental results show that our method outperforms prior works while achieving a low parameter count. We summarize our main contributions as follows:

- We present a new surgical scene graph-based VQA dataset, SSG-VQA, providing complex, diverse, geometrically grounded and surgical action-oriented question–answers.
- We present a surgical VQA model, SSG-VQA-Net, utilizing a novel scene-aware feature extraction strategy to obtain state-of-the-art performance.

## Methodology

### SSG-VQA dataset

In this section, we explain the SSG-VQA dataset generation process consisting of creating surgical scene graphs, designing a question engine with diverse templates and employing a sampling strategy to mitigate data imbalance and question–condition bias.

#### Scene graph generation

We build our SSG-VQA dataset using the publicly available CholecT45 [3], m2cai16-tool-locations [11] and CholecSeg8k [12] datasets. Specifically, we train a tool detection model [15] on m2cai16-tool-locations [11] and a semantic segmentation model [16] on CholecSeg8k [12] to extract bounding boxes of surgical objects, including surgical instruments and anatomies. Then, we build the surgical semantic scene graph using the detections. A surgical scene graph can be formulated as a set of nodes and edges, where the nodes represent surgical objects that contain a set of attributes, i.e., color, location and type, and edges represent the spatial and action relations among the objects. The spatial relations are calculated by comparing the centroid of objects, and the action relations are provided by the triplet annotations from CholecT45 [3]. Then, we leverage the generated scene graphs as input to a question engine, as described below, to generate diverse question–answer pairs. Note that to create a clean test set of question–answer pairs, we manually correct the bounding boxes and class labels of scene graphs in the test videos.

#### Question engine

The question engine, which is responsible for generating diverse questions with various categories, requires two inputs, i.e., scene graph and question templates. We use the CLEVR engine [7, 17] and extend it to the surgical context. Specifically, the question engine can change question templates’ parameters conditioned on the surgical scene graph to express diverse questions. For example, the question “what is the tool to the left of yellow anatomy?” can be formed by the template “what is the tool<*R*><*C*><*L*><*T*>?”, by replacing the parameters<*R*>,<*C*>,<*L*> and<*T*> into “to the left of,” “yellow,” “null” and “anatomy.” The questions are parameterized by five parameters, namely<*C*> (color),<*L*> (location),<*T*> (type),<*N*> (name) and<*R*> (relationship). In total, there are 40 question templates containing different types of questions, such as *querying object* (e.g., “what is the name of instruments to the left of the gallbladder?”), *querying attribute* ( e.g., “there is an object that is both to the left of the yellow thing and below the brown anatomy; what is its location?”), *querying relation* (e.g., “what is the action being performed?”), *confirming existence* (e.g., “is there a bipolar in the top-mid location?”) and *counting* (e.g., “how many instruments are in the bottom right?”).

Generated questions also fall into three categories depending on their complexity: *zero-hop*, *one-hop* and *single-and*. Each requires different visual reasoning steps to resolve. Specifically, solving these three types of questions involves the understanding of relations between zero, one or two surgical objects, respectively. Examples from each category are provided in supplementary material. The question engine allows us to freely control the complexity, length and number of questions per image.

#### Sampling and balancing

Here, we introduce the applied strategies to reduce the effect of class imbalance and question–condition bias during SSG-QA dataset construction. Surgical VQA is a multi-choice task, which mainly includes questions about the surgical objects. Therefore, an imbalance in the occurrence of surgical objects could lead to an imbalance in their class distribution. To address that, we sample the frame amounts based on the surgical phase presence labels from the Cholec80 dataset, instead of sampling evenly like Cholec80-VQA [8]. The criteria for including or excluding frames from our dataset is based on achieving a balanced distribution of phase labels. First, we identify overrepresented phases, marked by a redundant of frames. We then mitigate the imbalance by excluding frames from these overrepresented phases, such as “preparation” and “Calot triangle dissection.” The process continued until the dataset achieved a state of balance that we reach a predetermined, nearly equal number of frames across all phases. Also, to address the question–condition bias that the information is leaked out from poorly formulated questions, we consult the clinical expert and remove the question templates that may contain the question–condition bias, e.g., “how many livers are there?” which would have answer = 1 for all the cases. Then, these templates are excluded for question–answer generation, avoiding the question–condition bias. Since our SSG-VQA dataset is based on surgical scene graphs, we ask experts to manually review our dataset’s surgical scene graphs for the testing set, ensuring the generated questions and answers are accurate for the evaluation. Also, we consult the experts and design the rules for the question engine to eliminate poorly formulated questions. For example, it does not generate the counting questions when the ground truth answer is related to the anatomy. Therefore, we can eliminate the questions, such as “What is the location of the $<N>$?” with $<>$=gallbladder when there is no gallbladder in the scene. These processing strategies prevent question–condition bias and avoid generating degenerate question–answer pairs. The overall pipeline is shown in Fig. 2.

### SSG-VQA-Net

**Fig. 3:**
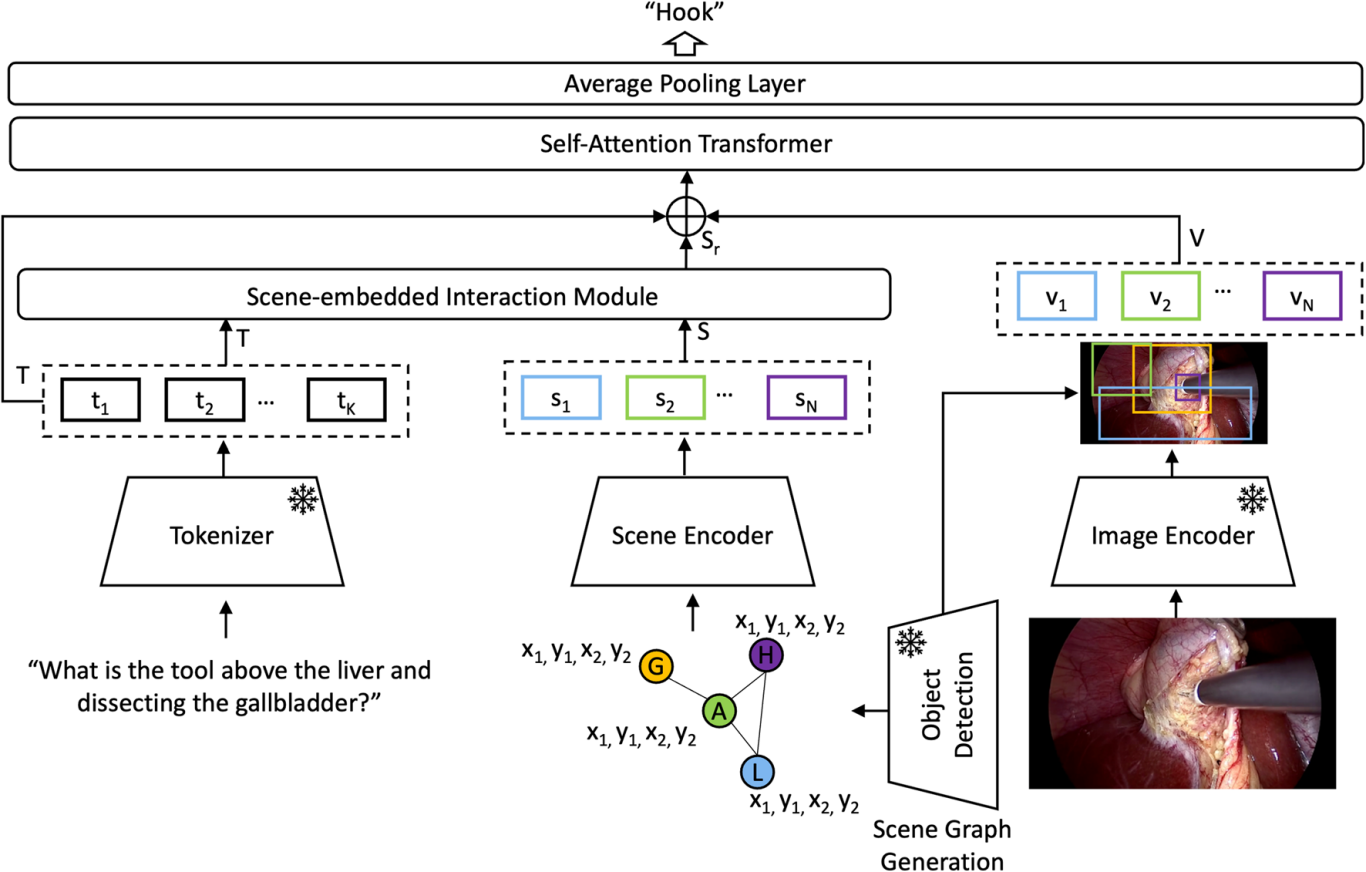
Pipeline of the SSG-VQA-Net. It requires three types of inputs, textual, visual and scene knowledge. The textual and scene embeddings are fed into the SIM and generate refined scene embeddings. The visual embeddings are generated from the RoIAlign. Finally, we concat them to feed into the self-attention transformer to get the final answer. Here, G, H, A and L represent class labels; $x_1$, $y_1$, $x_2$ and $y_2$ represent bounding box coordinates (G: gallbladder, H: hook; A: abdominal wall cavity; L: liver)

#### Pipeline

Here, we explain the pipeline of SSG-VQA-Net. Given the textual form of a question, we first extract textual embeddings of questions using a pretrained tokenizer [8], denoted as $T=\{t_1,\ldots t_{K}\}$. From the surgical scene image, we extract a feature map using the ResNet18 [18] visual backbone. Then we use a trained object detector, YOLOv7 [13], to detect the surgical objects and extract *N* object-wise visual embeddings using RoIAlign pooling, denoted as $V=\{v_1,\ldots v_{N}\}$. The object detector is trained on the bounding boxes of surgical objects from the SSG-VQA training dataset.

We build the scene embeddings using the detected surgical objects’ information. Specifically, we initialize the graph nodes as a concatenation of objects’ class labels and spatial coordinates, as shown in Fig. 3. These low-dimensional embeddings are projected using a linear layer, called Scene Encoder, to match the dimensionality of textual embeddings. These scene embeddings $S=\{s_1,\ldots s_{N}\}$ are then passed through our proposed Scene-embedded Interaction Module (SIM), explained below, to obtain text-aware scene embeddings ($S_r$). These text-aware scene embeddings ($S_r$) are then concatenated with the visual embeddings (*V*) and the textual embeddings (*T*) and passed through a self-attention-based transformer module. Finally, features are average-pooled and mapped to a predefined answer set to generate the output answer.

#### Scene-embedded Interaction Module

**Fig. 4:**
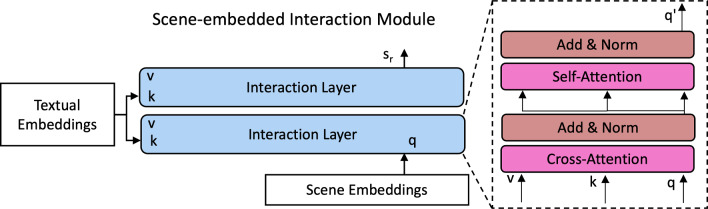
Scene-embedded Interaction Module. It is a stack of layers of cross-attention and self-attention. The cross-attention modulates the scene embeddings based on the text queries, while the self-attention refines the scene embeddings

In SSG-VQA-Net, initial scene embeddings *S* capture global surgical scene semantics. To handle complex questions that require localized focus, we introduce a lightweight Scene-embedded Interaction Module (SIM). The main objective of SIM is to correlate the textual embeddings with the scene embeddings. SIM consists of two interaction layers. Each layer comprises self-attention, cross-attention and feed-forward sub-layers, as shown in Fig. 4. The attention mechanism is defined as:

1 $$\begin{aligned} \text {Attention}(Q, K, V) = \text {softmax}\left( \frac{QK^T}{\sqrt{d_k}}\right) V, \end{aligned}$$

where the *Q* is the short for query, *K* is key and *V* is value.

In SIM, we first apply cross-attention, $S_r = \text {Cross-Attention}(S, T, T)$, to the textual and scene embeddings by processing textual embeddings *T* as key and value, and scene embeddings *S* as the query. This results in refined scene embeddings, which incorporate textual cues. Then, the refined scene embeddings are passed to the self-attention layer, $S_r = \text {Self-Attention}(S_r, S_r, S_r)$, to interact with themselves. By interacting with the textual embeddings and the scene embeddings, we obtain the textual-aware scene embeddings $S_r$. Through our ablation experiments, we show that the $S_r$ significantly contributes to providing correct answers to fine-grained questions.

**Table 1:** Dataset statistics comparison

Dataset	EndoVis-18-VQA [8]	Cholec80-VQA [8]	SSG-VQA
$\#$Surgical Scenes	2k	21k	25k
$\#$Questions	11k	43k	960k
$\#$Unique Questions	17	51	501k
Average Length in words	5.8	2.0	12.8
Average $\#$Questions per scene	5.0	6.5	38.9

We show that our dataset is more challenging and balanced as it includes more attributes and complexities in the questions

**Table 2:** Identification of question–condition bias in existing datasets

Methods	Endovis-VQA [8]			Cholec80-VQA [8]			SSG-VQA		
	Accuracy	Recall	F-score	Accuracy	Recall	F-score	Accuracy	Recall	F-score
L+Bert [19]	57.5	45.9	36.3	83.3	29.3	24.4	51.7	36.2	50.0
L+SciBert [20]	55.8	45.1	36.9	83.3	29.3	24.4	52.4	36.9	49.4
L+ClinicalBert [21]	60.4	**50**.**7**	**40**.**8**	83.1	29.4	24.4	52.3	35.4	49.9
VisualBert [22]	61.9	41.2	33.4	89.7	**62**.**9**	63.3	55.0	42.5	54.8
VisualBert Resmlp [22]	**63**.**2**	39.6	33.6	**89**.**8**	62.7	**63**.**4**	**58**.**7**	**44**.**8**	**57**.**7**

The best results are marked in bold

We use the accuracy, recall and F-score metrics from SurgicalVQA [8]. Endovis-VQA contains significant question–condition bias because the language model with pure language inputs can outperform the model with vision and language inputs

## Results and discussions

### Dataset comparison

SSG-VQA dataset contains the same train and test set videos as CholecT45 [3] dataset, which contains 40 laparoscopic cholecystectomy videos for training and 5 videos for testing. Our SSG-VQA dataset contains 960*k* questions from 25*k* surgical scenes. Table  1 presents the comparison between SSG-VQA and the typical datasets from prior work, i.e., EndoVis-18-VQA and Cholec80-VQA from [8], showing a $8\times$ and $22\times$ increase in number of questions, respectively. Also, our SSG-VQA dataset contains more diverse questions per scene (38.9 vs. 6.5) and much longer questions (12.8 words vs. 5.8 words). Furthermore, SSG-VQA contains a wider range of categories for object attributes, names and inter-object relationships, as shown in “Supplementary material.” Also, compared to the Cholec80-VQA dataset which provides 51 questions for all surgical scenes, our SSG-VQA has more diverse questions (501*k*) that are unique to surgical scenes, which prevents the model from overfitting to specific question patterns.

### Question–condition bias

VQA systems can exploit the question–condition bias from the dataset as a shortcut to answer questions without understanding the visual scenes. To quantify this bias, we train language-only models like ClinicalBert [21] to answer the questions without any visual information on existing VQA datasets, such as EndoVis-18-VQA and Cholec80-VQA, and on our SSG-VQA dataset. As shown in Table 2, the language-only model ClinicalBert outperforms the vision language multimodal models in EndoVis-18-VQA, suggesting the questions from EndoVis-18-VQA contain simple shortcuts to the correct answer. Cholec80-VQA and SSG-VQA have a lower bias as their questions are more vision-relevant. SSG-VQA further reduces bias by using scene graph-based diverse questions. In the following, we perform the experiments on the Cholec80-VQA and SSG-VQA dataset due to their low question–condition bias (Tables 3 and 4).

**Table 3:** Comparison results for baselines and our models

Models	Accuracy	mAP	Recall	F-score
L+ClinicalBert	52.3	40.4	35.4	49.9
VisualBert [22]	55.0	47.9	42.5	54.8
VisualBert Resmlp [8]	58.7	51.8	44.8	57.7
SurgicalGPT (RN18) [9]	*57.5*	*49.4*	*43.8*	*56.8*
SSG-VQA-Net	60.7	54.9	49.1	60.3
SSG-VQA-Net (Oracle)	**62**.**8**	**56**.**3**	**50**.**6**	**62**.**3**

The best results are marked in bold

The SSG-VQA-Net with scene knowledge achieves the best results. The SSG-VQA-Net (oracle) model refers to the model that uses detection labels from the SSG-VQA dataset to construct the scene embeddings instead of using the trained YOLOv7 object detector

**Table 4:** Breakdown results of the prior models and our models

	VisualBERT [22]	VisualBERTMLP [8]	SSG-VQA-Net	SSG-VQA-Net (oracle)
Query object	39.4	38.4	48.0	**55**.**4**
Query attribute	51.7	54.5	54.8	**60**.**2**
Existence	68.0	**76**.**4**	73.9	72.7
Counting	24.5	29.6	**36**.**9**	24.2
Zero-hop	50.4	53.2	**56**.**6**	55.0
One-hop	46.4	46.2	50.3	**51**.**9**
Single-and	23.4	30.9	39.0	**41**.**4**

The best results are marked in bold

We show that our model outperforms the baselines by a large margin, especially on the complex questions that require visual reasoning. Also, the results on a different set of questions show that our dataset is not dominated by one type of question. We report the mAP here

### Detection and segmentation models

**Table 5:** Performance of the detection and segmentation models that we use for SSG-VQA dataset construction

Metrics						
*(a) The performance of the detection model that we use to construct SSG-VQA dataset. We report the instrument detection performance of the trained detection model*						
Precision	0.9521					
Recall	0.9358					

Class	Abdominal cavity	Liver	Gut	Omentum	Gallbladder	Cystic Duct
*(b) The performance of the detection model that we use to construct SSG-VQA dataset. We report the dice over the anatomical classes*						
Dice	0.8477	0.9422	0.4369	0.8658	0.7436	0.0146

We train YOLO model on m2cai16-tool-locations dataset and DeepLab model on CholecSeg8K dataset to construct the SSG-VQA dataset. Specifically, we apply the trained models on the CholecT45 dataset to generate pseudo labels, i.e., segmentations for the anatomy and the bounding boxes for the tools. The segmentation outputs are further converted to the bounding boxes. We use these bounding boxes to construct the scene graphs and generate the question–answer pairs. As shown in Table 5b, we find that the trained YOLO model is adept at detecting surgical instrument objects. The model demonstrates high precision, suggesting a low rate of false positives. This efficiency in detecting instruments shows the model’s strength to construct high-quality SSG-VQA dataset. Also, as shown in Table 5b, the segmentation model trained on CholecSeg8K can segment various anatomical objects, including the abdominal wall cavity, liver, gut, omentum, gallbladder and cystic duct. It demonstrates the segmentation model’s ability to accurately segment most of the anatomy classes. However, a notable exception was observed in the segmentation of the cystic duct.

### Results of SSG-VQA-Net

#### Results on SSG-VQA

**Comparison to other works**. As shown in Table 3, SSG-VQA-Net outperforms baseline models like VisualBert [22] and VisualBert Resmlp [8] in metrics such as mAP, recall and F-score. We also train an upper bound model, called SSG-VQA-Net (oracle), that uses the scene embeddings from detection labels of the SSG-VQA dataset instead of using the trained YOLOv7 object detector. This model outperforms prior works significantly, emphasizing the importance of high-quality scene embedding inputs. Additionally, we train the SurgicalGPT on our SSG-VQA dataset to show the effect of a strong language decoder. Specifically, we fix the feature extraction process of SurgicalGPT to be the same as VisualBert, ensuring fair comparison. Our model achieves superior results, demonstrating the feature extraction process and additional scene graph knowledge are more beneficial than sequence decoding.

**Analysis by question type**. As shown in Table 4, SSG-VQA-Net can handle various question types. For “counting” questions, it outperforms VisualBert by 7.3 points in mAP. For “existence” and “query object” types, the model again shows superior performance w.r.t to baseline models.

**Analysis by complexity**. SSG-VQA dataset provides the diagnostic setup to pinpoint the weakness of the model. As shown in Table 4, we compute the performance of our models on questions that require different visual reasoning complexity, i.e., *zero-hop* and *one-hop*, and *single-and*. Our model shows consistent gains in both simple and complex question queries. For *one-hop* and *single-and* type of questions, SSG-VQA-Net achieves a 4.1 and 8.1 point mAP increase over VisualBERT ResMLP, respectively. This indicates that the inclusion of scene context can aid in resolving complex queries.

#### Results on Cholec80-VQA

**Table 6:** Results on the Cholec80-VQA

	#Parameter	Accuracy	Recall	F-score
VisualBert [22]	209.8M	89.7	62.9	63.3
VisualBert Resmlp [8]	184.7M	89.8	62.7	63.4
SurgicalGPT (RN18) [9]	234.5M	87.5	57.5	57.9
*SurgicalGPT (ViT)* [9]	*309.5M*	*92.3*	*68.3*	*69.6*
*SurgicalGPT (Swin)* [9]	*312.5M*	*94.3*	*73.4*	*74.4*
SSG-VQA-Net	145.3M	**90**.**6**	**64**.**4**	**63**.**7**

The best results are marked in bold

SSG-VQA-Net achieves better results than the state-of-the-art models, even w.r.t SurgicalGPT, which contains a heavy sequence decoding module of GPT-2

We also conduct the experiments on the other publicly available surgical VQA dataset Cholec80-VQA. As illustrated in Table 6, SSG-VQA-Net significantly outperforms the SurgicalGPT [9], which requires heavy sequence decoding using GPT-2 architecture. This highlights that the bottleneck of the current surgical VQA problem lies in the visual scene understanding instead of text generation. Also, even using YOLOv7 for object detection, our model achieves higher performance with fewer parameters than prior works, verifying its efficiency while maintaining higher performance metrics.

Our final model, SSG-VQA-Net, uses ResNet18 as its backbone instead of higher-capacity ViT and Swin Transformers to balance the performance and the efficiency. As shown in Table 6, SSG-VQA-Net significantly reduces parameter count by about half compared to ViT and Swin versions of SurgicalGPT, while still delivering strong results.

#### Ablation study

**Table 7:** Effect of different modules

SIM	ROI	Accuracy	mAP	Recall	F-score
$\times$	$\times$	55.0	47.9	42.5	54.8
$\checkmark$	$\times$	57.1	51.0	47.2	56.1
$\times$	$\checkmark$	58.8	52.5	47.9	57.7
$\checkmark$	$\checkmark$	**60**.**7**	**54**.**9**	**49**.**1**	**60**.**3**

The best results are marked in bold

RoIAlign pooling boosts results, and the Scene-embedded Interaction Module further enhances them. Both modules offer complementary benefits

Table 7 shows that combining both the Scene-embedded Interaction Module (SIM) and RoIAlign (ROI) pooling significantly boosts the model’s performance. This suggests that these modules are not just individually beneficial but are actually complementary. Specifically, the model attains the highest mAP (54.9%) when both components are added. Also, the improvement indicates that introducing scene knowledge representation learning is crucial for robust surgical visual question answering.

## Conclusion

In this paper, we tackle the problem of visual question answering (VQA) in the context of fine-grained surgical scene understanding. First, we introduce a new dataset called SSG-VQA, which uses a surgical scene graph as an underlying representation and a question–answer generation engine to generate diverse, geometrically grounded and surgical action-oriented question–answer pairs. The question–answer pairs are also sampled to mitigate the question–condition bias that exists in the current surgical VQA datasets. We also propose a novel model called SSG-VQA-Net to explicitly incorporate scene knowledge and object-wise local features in the VQA model design to improve the reasoning ability on complex questions. The results show that SSG-VQA-Net outperforms existing baseline models by a large margin.

### Supplementary Information

Below is the link to the electronic supplementary material.

Supplementary file 1 (png 1054 KB)

Supplementary file 2 (png 1153 KB)

Supplementary file 3 (png 5521 KB)

Supplementary file 4 (png 1110 KB)

Supplementary file 5 (png 2369 KB)

Supplementary file 6 (png 822 KB)

Supplementary file 7 (png 425 KB)

Supplementary file 8 (jpg 135 KB)

Supplementary file 9 (png 1883 KB)

Supplementary file 10 (tex 12 KB)
