# Supplementary figures and images for: Advancing surgical VQA with scene graph knowledge
Source: Int J Comput Assist Radiol Surg. 2024 May 23;19(7):1409–17. doi: 10.1007/s11548-024-03141-y (PMC11231006; doi:10.1007/s11548-024-03141-y)

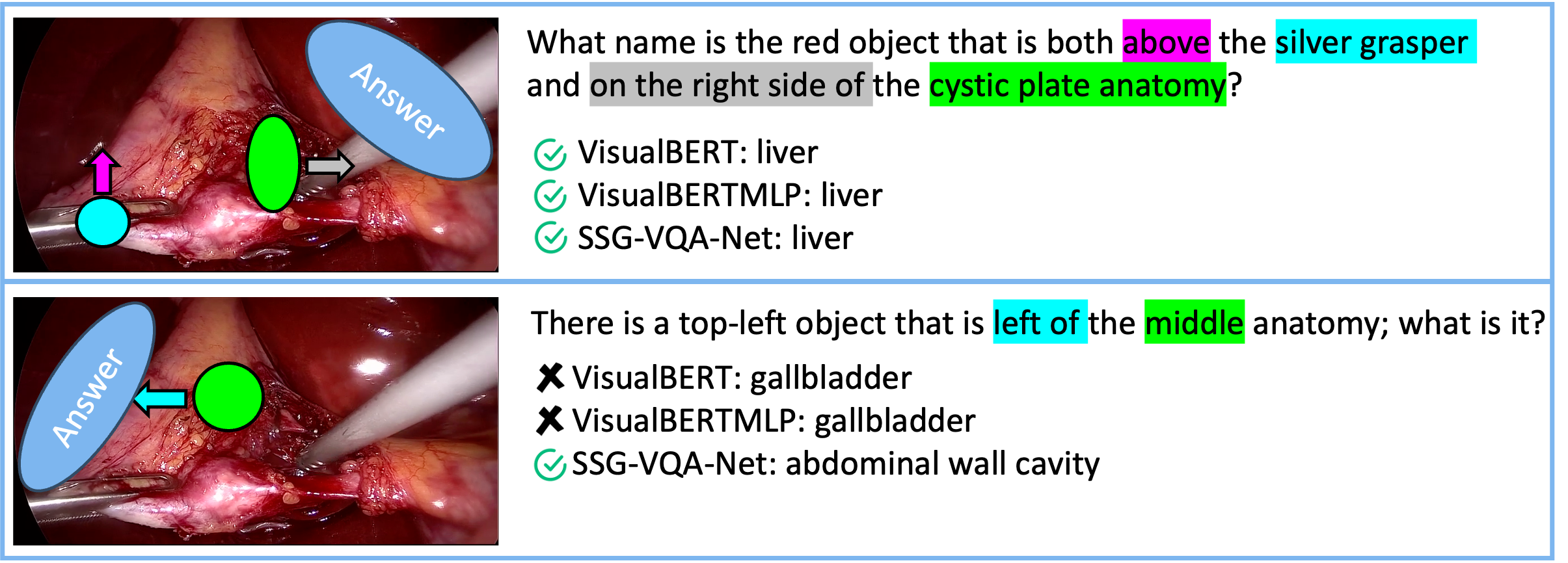

Supplement: Supplementary file 1 — (png 1054 KB) [file 11548_2024_3141_MOESM1_ESM.png]

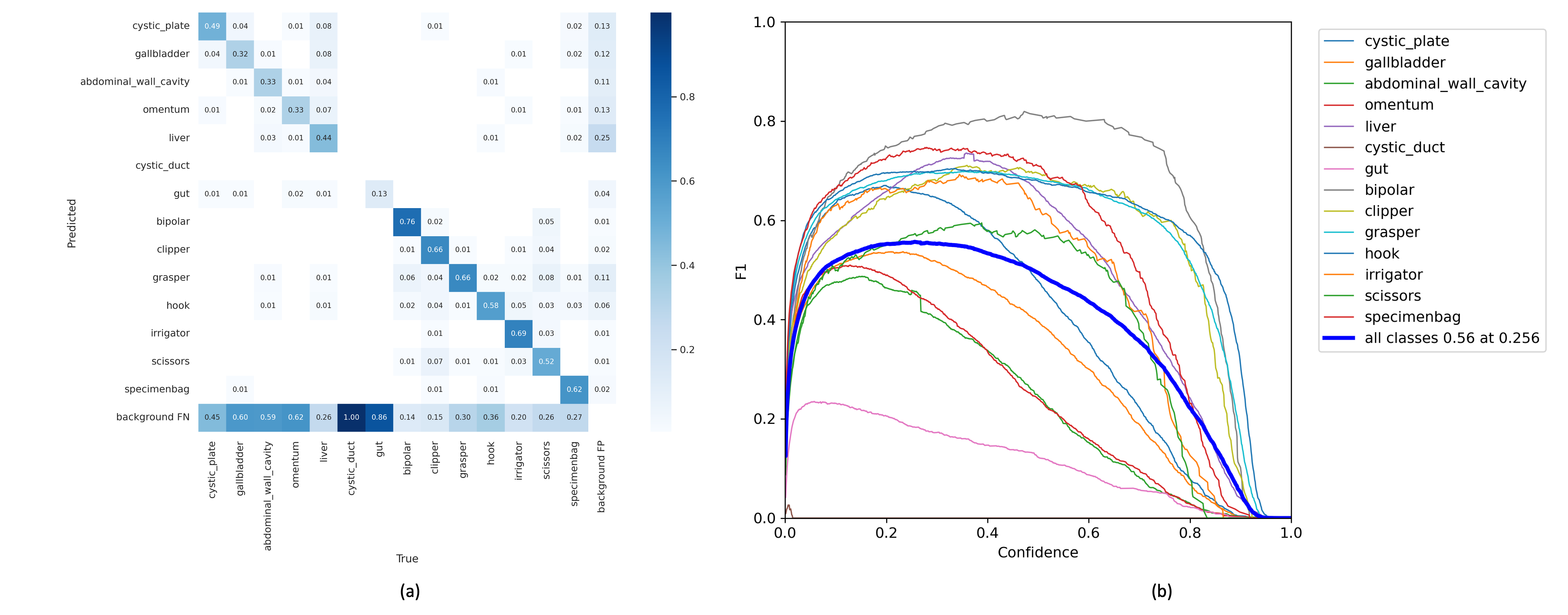

Supplement: Supplementary file 2 — (png 1153 KB) [file 11548_2024_3141_MOESM2_ESM.png]

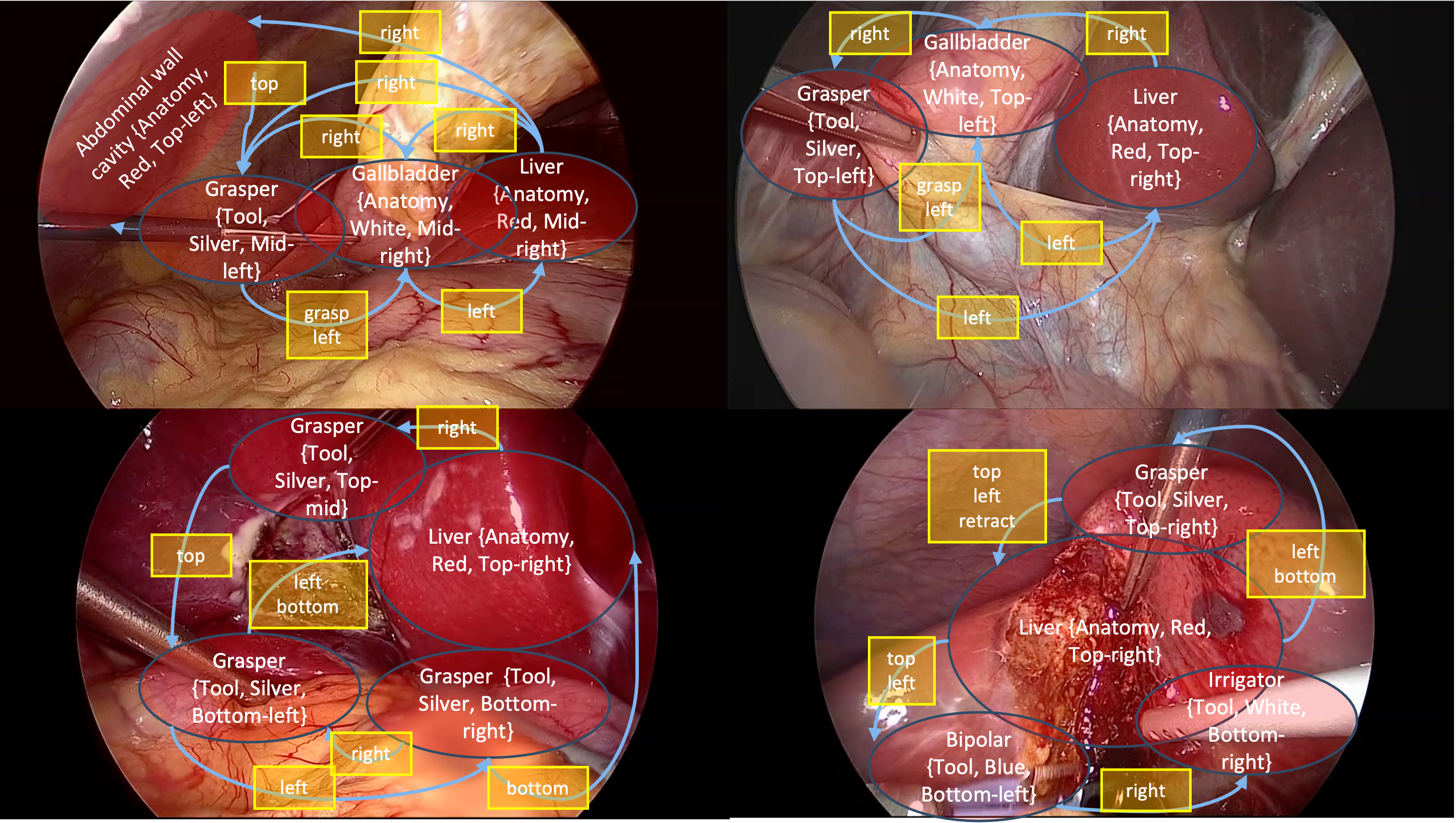

Supplement: Supplementary file 3 — (png 5521 KB) [file 11548_2024_3141_MOESM3_ESM.png]

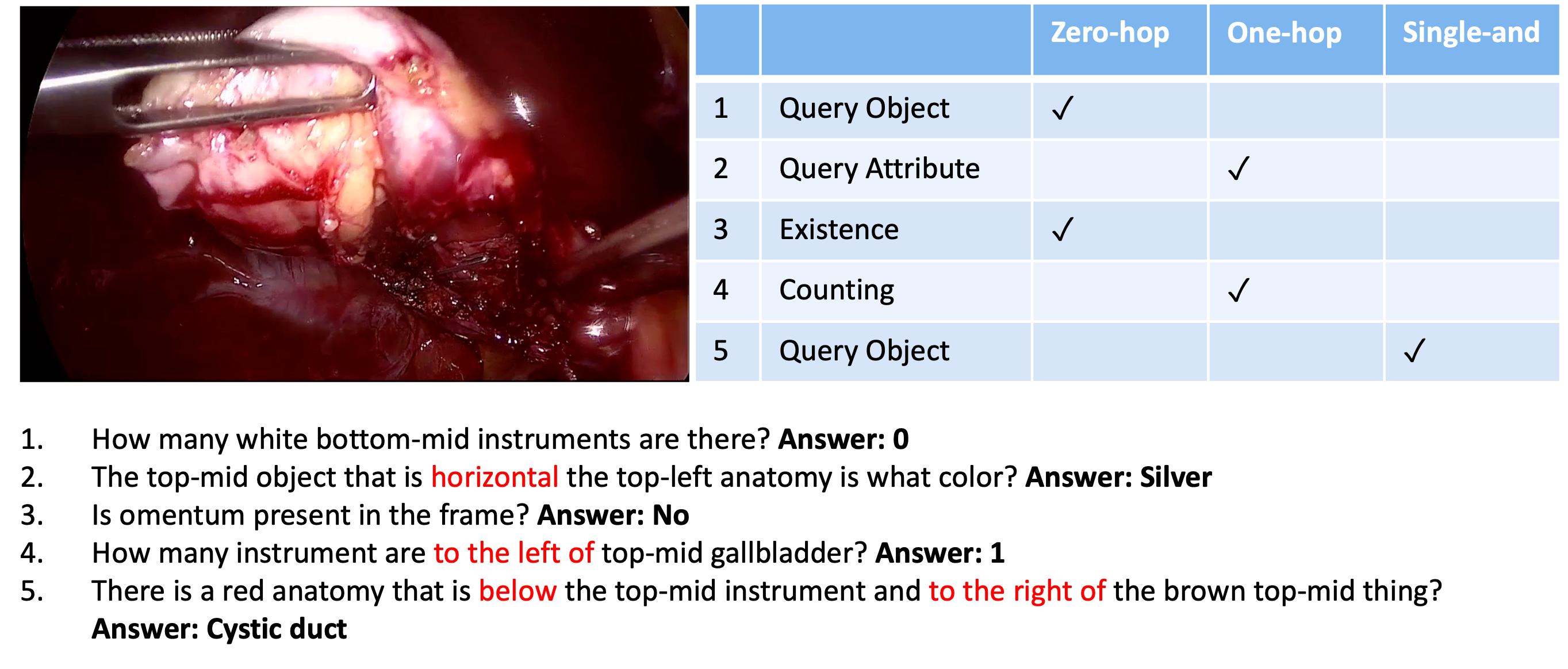

Supplement: Supplementary file 4 — (png 1110 KB) [file 11548_2024_3141_MOESM4_ESM.png]

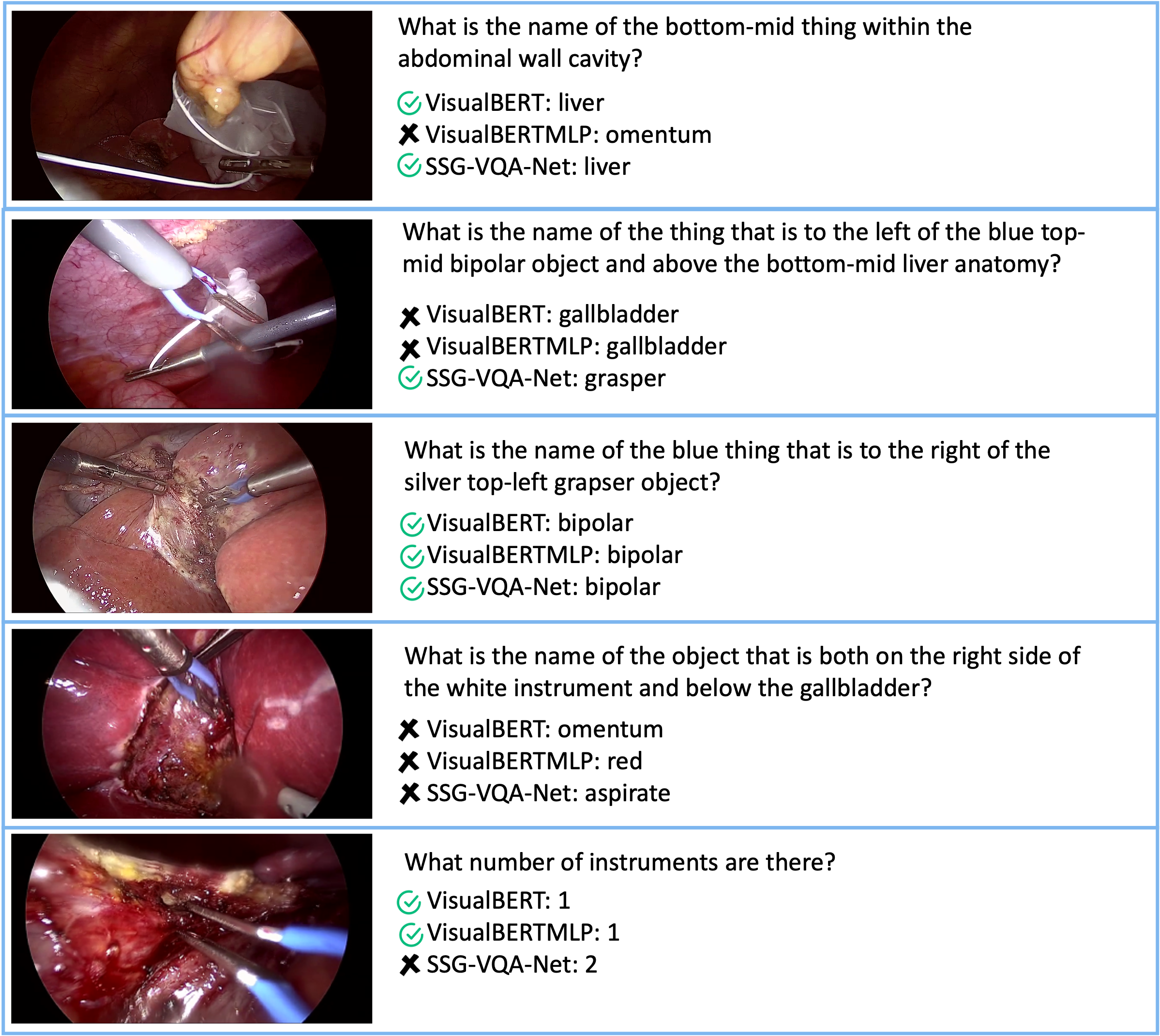

Supplement: Supplementary file 5 — (png 2369 KB) [file 11548_2024_3141_MOESM5_ESM.png]

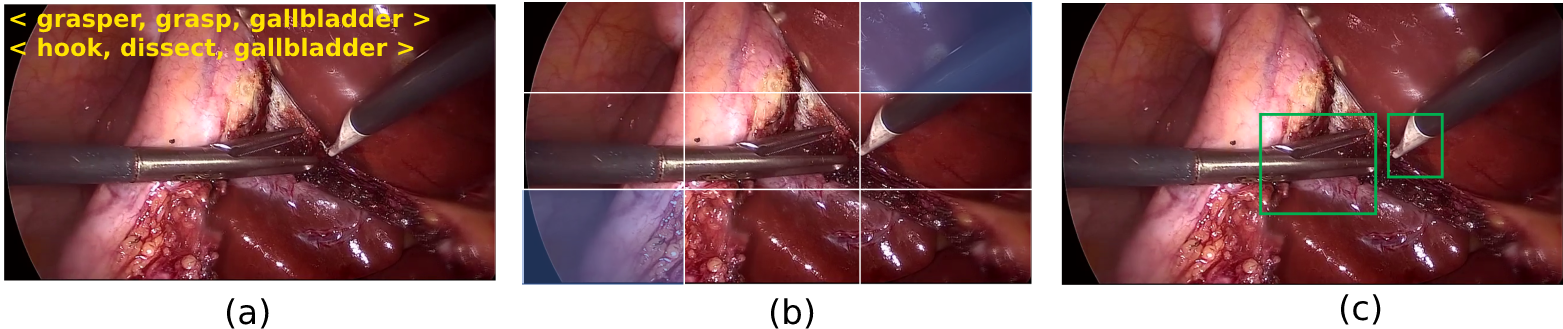

Supplement: Supplementary file 6 — (png 822 KB) [file 11548_2024_3141_MOESM6_ESM.png]

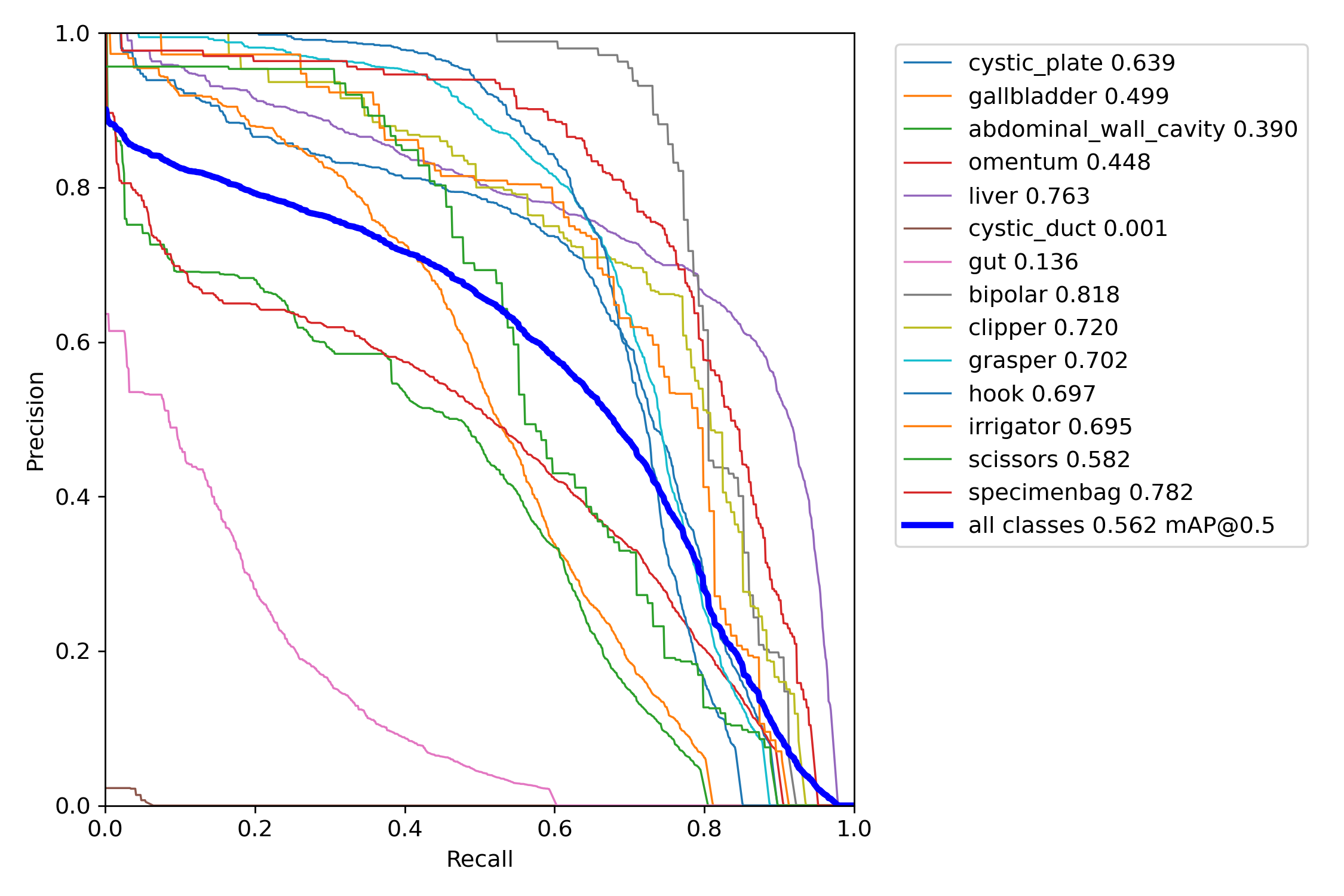

Supplement: Supplementary file 7 — (png 425 KB) [file 11548_2024_3141_MOESM7_ESM.png]

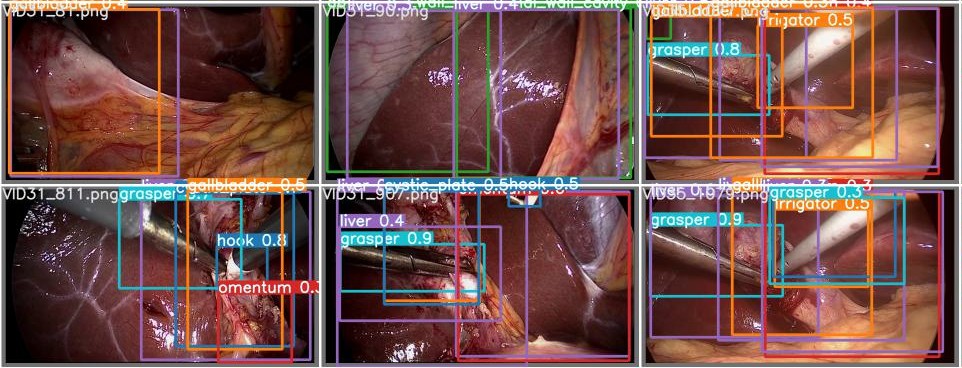

Supplement: Supplementary file 8 — (jpg 135 KB) [file 11548_2024_3141_MOESM8_ESM.jpg]

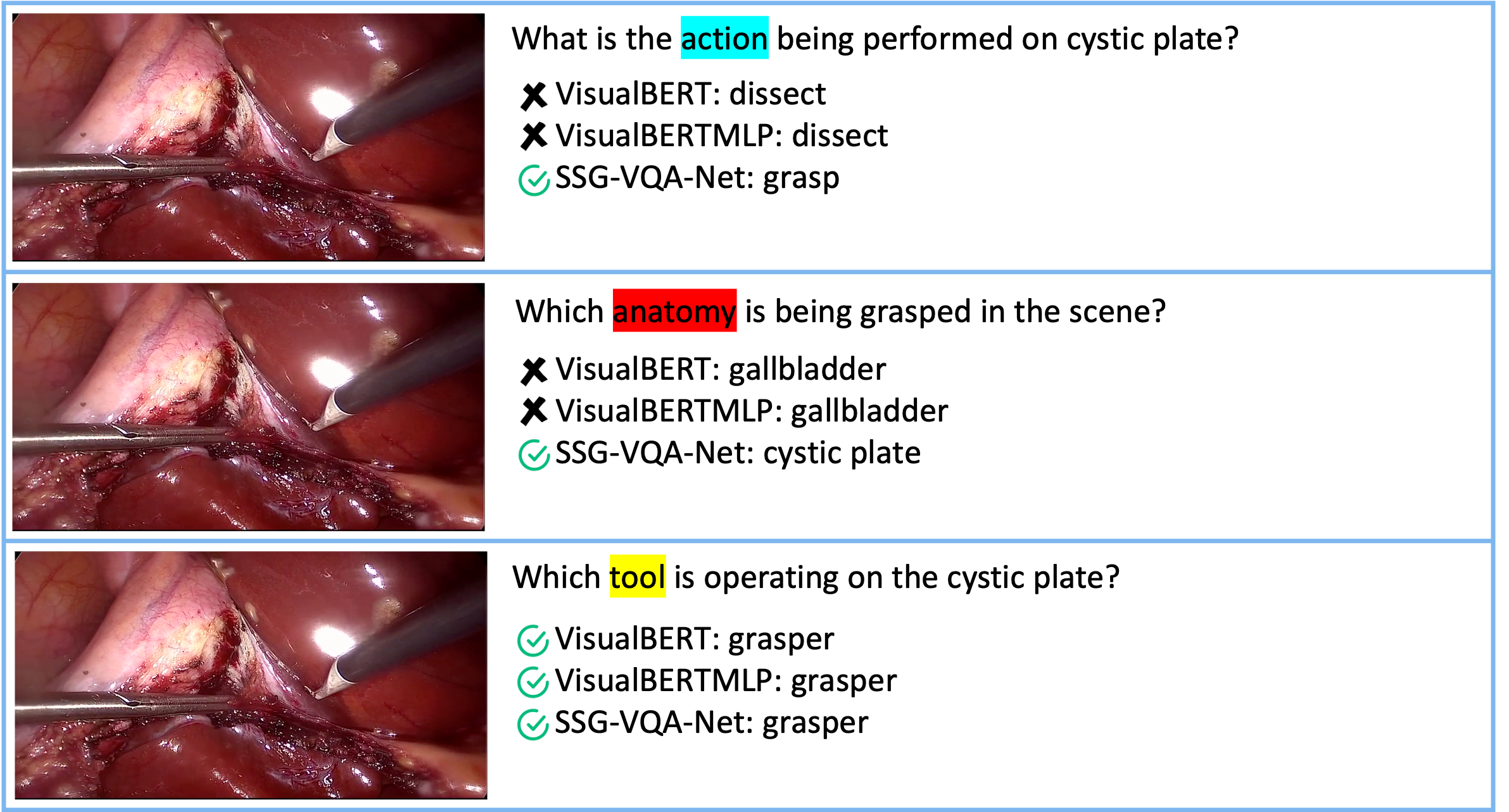

Supplement: Supplementary file 9 — (png 1883 KB) [file 11548_2024_3141_MOESM9_ESM.png]
